# Special Issue “Molecular Research in Breast Cancer: Pathophysiology and Treatment”

**DOI:** 10.3390/ijms26199732

**Published:** 2025-10-07

**Authors:** Costa Frangou

**Affiliations:** Department of Cell and Cancer Biology, University of Toledo College of Medicine and Life Sciences, 3000 Arlington Avenue, Toledo, OH 43614, USA; costakis.frangou@utoledo.edu; Tel.: +1-(467)-383-4168

## 1. Introduction

Breast cancer remains the most commonly diagnosed malignancy in women and a leading cause of cancer-related mortality worldwide [1], but scientific and technological advances over the past two decades have transformed our understanding of this complex disease, with advances in multi-omic profiling shaping our view of breast cancer as a dynamic ecosystem. Furthermore, we now recognize that breast cancer is not a single, static disease but instead a family of evolving states shaped by genotype, cellular plasticity, and the microenvironment. While these insights inform classification and treatment strategies, they also underscore a daunting challenge: achieving durable disease control. Specifically, tumor heterogeneity drives adaptation, which in turn promotes therapy resistance and relapse mechanisms [2,3]. The overarching challenge for precision oncology is to translate these molecular insights into actionable, decision-quality information at the bedside—identifying which patients will benefit from which treatments and when.

## 2. Advances in Molecular Pathophysiology

Comprehensive cancer genome sequencing and integrative computational analyses have systematically mapped the (epi)genetic landscapes of breast cancer, defined distinct tumor subtypes [4,5], and identified novel tumor subgroups that inform the stratification of risk assessment and therapeutic strategies. The recent advent of single-cell and spatial technologies enables the in situ resolution of epithelial, stromal, and immune programs, connecting tumor cells to their neighborhoods, niches, and clinical behaviors [6]. Notably, these approaches move beyond bulk averages to track minority populations, such as stem-like, therapy-tolerant, and immune-evasive states that often determine patient outcomes [7], while showing how biochemical, mechanical, and microenvironmental cues modulate oncogenic signaling and cell fate outcomes.

Cancer genome studies and functional analyses have identified recurrent cancer drivers and begun to identify the regulatory axes that integrate diverse oncogenic signals [8]. Emerging mechanobiology research suggests that the mechanics of the nuclear and extracellular matrix influence tumor cell invasion, DNA damage responses, and immune interactions [9,10]. Incorporating these physical cues into therapeutic design could expand the potential for durable disease control. Context-sensing pathways such as YAP/TAZ–TEAD intersect with hormone receptor and HER2 signaling programs to modulate cell proliferation, survival, and plasticity in varying ways across tissues and cellular states [11]. Collectively, these advances establish a causal link between cancer driver genotype, cell state, microenvironment, and therapeutic response, suggesting the potential to design state-selective therapeutic interventions rather than lineage-agnostic ones.

## 3. Persistent Bottlenecks: Interconnected Challenges That Hinder Translation

Tumor heterogeneity and plasticity remain the primary uncertainties in predicting who will benefit from a given therapy, for how long, and how the disease will adapt. Intratumoral diversity and dynamic state transitions complicate these predictions, because the unit of selection is not a single tumor clone but an evolving distribution of cell states that can reprogram in response to therapy [12]. Consequently, snapshots taken at diagnosis are often inadequate guides to the trajectories that become relevant many months later. Without longitudinal, state-aware measurements, treatment plans risk targeting ‘yesterday’s disease.’

Resistance mechanisms are multifactorial and complex. Tumors frequently evade targeted treatments and chemotherapy by reactivating compensatory signaling pathways, epigenetic reprogramming, or niche-mediated protection. Clinical studies such as PALOMA-3 show how selective pressure shapes the cancer genome and epigenome, producing distinct resistance archetypes that call for tailored counterstrategies [13]. To overcome resistance, therapies must be designed with anticipated escape routes in mind, guided by early pharmacodynamic (PD) readouts rather than delayed radiographic changes.

Biomarker development and implementation lag behind the discovery of molecular targets. Although many candidate biomarkers exist [14,15], few demonstrate consistent, cross-site performance in clinical settings. Despite this lack, several analytically validated biomarkers guide routine decision-making in breast oncology. For example, tissue immunohistochemistry for ER/PR and HER2, PD-L1 testing in metastatic triple-negative disease, and companion diagnostic-linked genomic assays determine eligibility for endocrine, HER2-directed, immuno-, and targeted therapies. Multigene expression signatures, such as Oncotype DX, MammaPrint, EndoPredict, Prosigna/PAM50, and Breast Cancer Index, refine decisions on adjuvant chemotherapy and extended endocrine therapy in early HR+/HER2− breast cancer. Nevertheless, cross-assay and cross-site variability—illustrated by PD-L1 clone discordance and evolving HER2-low reporting—underscore the need for rigorous validation, the use of FDA-approved companion diagnostics when available, and harmonized performance standards across laboratories.

Metastatic disease remains largely incurable. Organ-specific metastasis reflects distinct ecological rules across different tissues, and dormancy and late relapse indicate state-dependent survival and reactivation programs that are poorly understood or in many instances remain enigmatic [16,17]. At the other end of the spectrum, early detection of breast cancer demands identifying the disease as soon as possible, ideally when it is small and localized to the breast. Although mammography saves lives, its broad use raises concerns about overdiagnosis and the overtreatment of indolent lesions [18], so the field faces a twin challenge: distinguishing aggressive from low-risk disease early and accurately while, conversely, preventing or containing the lethal biology of metastasis. Meeting these challenges requires new experimental models and measurements that longitudinally resolve tumor cell states in patient-relevant contexts. In parallel, improved models of organotropism are urgently needed to explain why certain subtypes metastasize to different tissues and identify niche-specific vulnerabilities that therapies can exploit.

## 4. Routes to Accelerate Translation

Recognizing that progress can accelerate along multiple avenues, we highlight a focused set of priorities that can be rapidly implemented. For brevity, we selected four domains that offer feasibility and near-term impact: interoperable, longitudinal, patient-proximal profiling; microenvironment-aware therapies and endpoints; adaptive trial architectures anchored by early PD decision gates; and standards and equity as enabling infrastructure. These are summarized below.

(1) Develop interoperable, longitudinal, patient-proximal profiling systems by integrating baseline multi-omics data (genome, transcriptome, proteome) with repeated, minimally invasive sampling during therapy, such as circulating tumor DNA and single-cell or spatial biopsies. Align these data with standardized PD endpoints. Patient-derived organoids and xenografts should serve as ‘shadow trials’ that evaluate treatment combinations based on the patient’s evolving state rather than on a static genotype. Harmonizing metadata—including treatments, timing, and clinical covariates—will enhance the reusability and comparability of these datasets across cancer centers.
(2) Design therapies and clinical trial endpoints that consider the tumor microenvironment. Unlike approaches targeting only tumor-intrinsic factors, interventions should explicitly address immune and stromal programs that support tumor persistence, such as macrophage polarization, T-cell exclusion, and extracellular matrix remodeling. Clinical trials can incorporate microenvironmental PD markers—such as immune infiltration, spatial proteomics, and matrix signatures—alongside tumor cell readouts to confirm on-target effects within the tumor ecosystem. Combining state-selective tumor agents with immune or stromal modulators early in treatment (before resistance is established) offers a feasible, promising strategy.
(3) Modernize trial architecture to improve adaptability. Basket and umbrella clinical trial designs, adaptive randomization, and early PD-based go/no-go criteria are well suited to state-aware oncology. Within this framework, the dynamics of circulating tumor DNA, single-cell signatures of stress or lineage switching, and pathway-specific PD markers can mark interim decision points, mitigating risk when a regimen deviates from its expected trajectory. Crucially, biomarker assays must be analytically validated, with predefined action thresholds and standardized performance metrics, to ensure consistency across sites.
(4) Commit to universal standards and equity as essential scientific enablers. Standardized nomenclature, assay calibration, and reporting templates are not bureaucratic burdens but fundamental scientific infrastructure; they enhance the signal-to-noise ratio and reveal true effects across heterogeneous populations. Technical variability, small training datasets, shifting definitions, and limited analytical validation hinder their broad adoption. In practice, these challenges undermine precision oncology efforts by reducing confidence in the determination of which breast cancer patients should receive a given therapy and when to stop or switch treatments. Similarly, equitable access to testing and targeted therapies is integral to valid inference; uneven access biases both the benefits and the evidence base. Translational studies should consider prospective implementation metrics—such as turnaround time, uptake, and coverage—as primary outcomes that are as significant as response and survival.

## 5. Conclusions

Breast cancer research has rapidly progressed beyond one-size-fits-all treatments. The overarching challenge of breast cancer research is translating fundamental molecular insights into knowledge that guides and improves patient care. Achieving this goal will demand the development of interoperable longitudinal datasets linked to clinical outcomes, early PD markers that validate underlying mechanisms, clinical trial designs incorporating robust decision gates, and standardized assays that can be reliably used across multiple sites. At the same time, equitable access to diagnostic testing and targeted therapies must be intentionally designed and rigorously measured, as disparities in access lead to unequal patient benefits. We should routinely evaluate tumor-intrinsic agents alongside microenvironment-modulating strategies when biological evidence warrants it and should invest in patient-proximal models that capture the immune and stromal context. We must also develop analytical tools that effectively summarize tumor adaptation and evolution in clinically actionable terms.

This editorial presents a practical, albeit not exhaustive, framework that research teams can employ immediately to prioritize measurements, endpoints, and trial designs, facilitating the translation of molecular insights into earlier, more durable, and more equitable patient benefits without overstating the current evidence. In this context, we hope that this special issue—“Molecular Research in Breast Cancer: Pathophysiology and Treatment”—provides a valuable resource for disseminating basic and translational research and fostering productive interdisciplinary dialogue. Our shared objective is to hasten progress toward more effective and lasting treatments for patients with breast cancer.
